# Physiological Roles of GPR10 and PrRP Signaling

**DOI:** 10.3389/fendo.2013.00020

**Published:** 2013-03-05

**Authors:** Garron T. Dodd, Simon M. Luckman

**Affiliations:** ^1^Faculty of Life Sciences, AV Hill Building, University of ManchesterManchester, UK

**Keywords:** PrRP, GPR10, energy intake, stress, dorsomedial hypothalamic nucleus, nucleus tractus solitarius, energy metabolism

## Abstract

Prolactin-releasing peptide (PrRP) was first isolated from bovine hypothalamus, and was found to act as an endogenous ligand at the G-protein-coupled receptor 10 (GPR10 or hGR3). Although originally named as it can affect the secretion of prolactin from anterior pituitary cells, the potential functions for this peptide have been greatly expanded over the past decade. Anatomical, pharmacological, and physiological studies indicate that PrRP, signaling via the GPR10 receptor, may have a wide range of roles in neuroendocrinology; such as in energy homeostasis, stress responses, cardiovascular regulation, and circadian function. This review will provide the current knowledge of the PrRP and GPR10 signaling system, its putative functions, implications for therapy, and future perspectives.

## Introduction

Seven-transmembrane-domain receptors (7TMRs) make up a receptor superfamily related by common signaling features and a structure that spans the cell membrane seven times. All 7TMRs are coupled to guanine nucleotide binding proteins (G-proteins) and, as such, are more commonly referred to as G-protein-coupled receptors (GPCRs; Probst et al., 1992). In the human genome, over 800 GPCRs have been annotated (>4% of the genome), many of which since have been implicated in diverse physiological roles from photoreception to olfaction, and from mood to appetite (Fredriksson et al., 2003). This diverse functionality infers immense therapeutic potential for the treatment of disease and, in fact, as many as half of the currently marketed drugs target GPCRs (Flower, 1999). Advances in genomics over the last century, that have allowed genome-wide homology analysis, have facilitated the discovery of so many new GPCRs. Currently the GenBank/EMBL database has over 1000 clones of eukaryotic GPCRs recorded, and many of the predicted receptors have no known ligand. These are termed “orphan” GPCRs. Although many of the GPCR genes probably correspond to homologs of sensory olfactory receptors, which are predicted to exist in considerable number in the genome, the remainder could encode for diverse unknown receptors, which may play important physiological roles (Buck and Axel, 1991). Due to the undoubted therapeutic potential for the treatment of different pathologies, the discovery of ligands by the “de-orphanization” of GPCRs and an understanding of their physiological function is the focus of an intense research effort that has far reaching implications for both frontier and translational science.

One of the first GPCRs to be de-orphanized was G-protein-coupled receptor 10 (GPR10; also known as hGR3 or UHR-1). GPR10 was originally cloned in hypothalamic tissue using low stringency PCR primers designed against to the highly conserved GPCR transmembrane domains 2 and 6 (Welch et al., 1995). The cloned receptor showed sequence similarity to the neuropeptide Y (NPY) receptor (31% overall and 46% in the transmembrane regions), however, it could not be activated by either NPY or pancreatic polypeptide (Marchese et al., 1995). This presented the scientific community with a novel problem, in that this represented the first GPCR for which its discovery preceded that of its endogenous ligand. Initial GPR10 localization studies indicated high mRNA expression in the anterior pituitary (Fujii et al., 1999). As hypothalamus derived factors frequently play important roles in regulating anterior pituitary function, it seemed intuitive that the natural ligand for GPR10 might exist in the hypothalamus. Using this insight, GPR10 was finally de-orphanized by Hinuma et al. (1998), using a novel reverse pharmacology approach. For reasons described below, the receptor ligand was termed prolactin-releasing peptide (PrRP). Later studies, using other *in vitro* heterologous expression systems, demonstrated that PrRP shows some promiscuous binding to another RFamide peptide family receptor, neuropeptide FF receptor 2 (NPFF-2R) (Engstrom et al., 2003; Ma et al., 2009). However, to date, PrRP is the only ligand known to have significant affinity for GPR10.

Initial studies showed that PrRP could stimulate prolactin secretion from dispersed anterior pituitary cells; hence, the peptide’s name (Hinuma et al., 1998). However since its discovery, the importance of PrRP in the physiological regulation of prolactin secretion has been put in doubt (see below). Instead, the PrRP-GPR10 signaling pathway has been implicated in a range of other physiological systems. For example, central administration of PrRP inhibits food intake and increases energy expenditure in rats and mice (Lawrence et al., 2000, 2004), suggesting that PrRP plays roles in the regulation of energy balance. It also elevates circulating plasma levels of adrenocorticotropic hormone (ACTH) level, suggesting an association of PrRP with stress responses (Takayanagi and Onaka, 2010). Moreover, PrRP also can affect the cardiovascular system (Samson et al., 2000) and circadian cyclicity (Zhang et al., 2000, 2001; Lin et al., 2002a). This article aims to review the current understanding of the physiological roles for PrRP and GPR10 signaling in the mammalian system, and to highlight future directions for research.

## PrRP and GPR10 Expression

**Figure 1:**
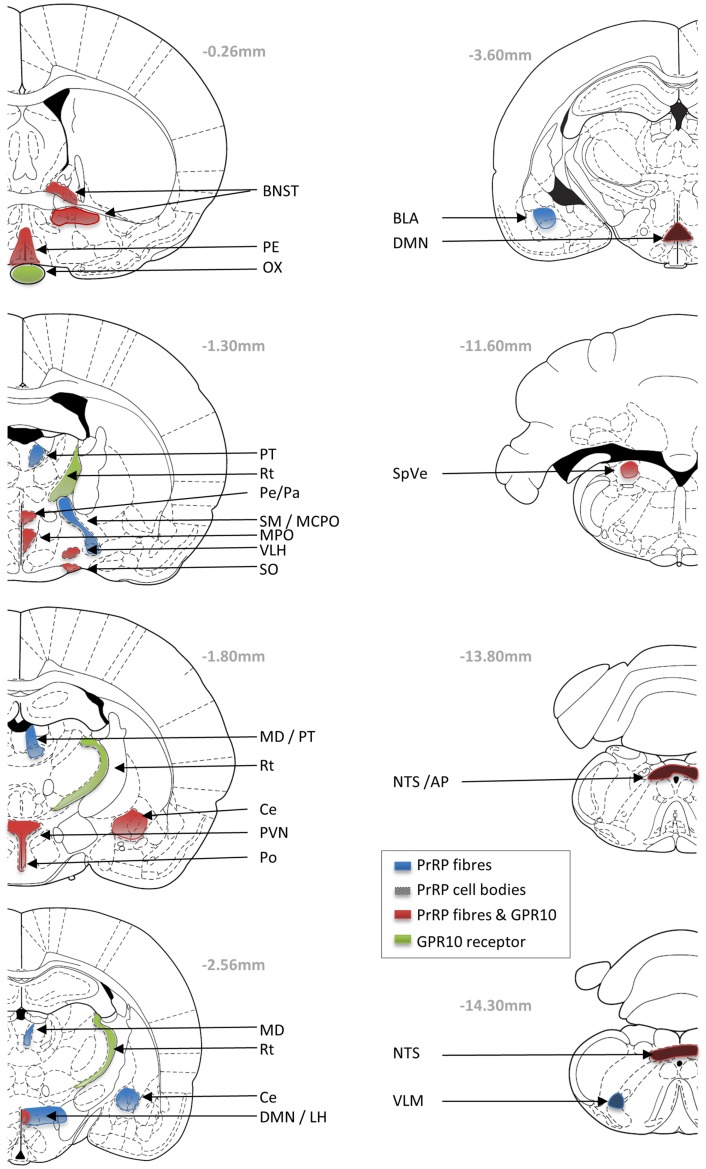
**Schematic drawings showing the neuronal distribution of PrRP and GPR10 receptor in the Paxions and Watson rat brain atlas (Paxinos and Watson, 1998; Sun et al., 2005)**. Blue areas represent PrRP-immunopositive nerve fibers; black checkers represent PrRP cells bodies; green areas represent GPR10 expression; and red areas represent overlap of PrRP and GPR10 expression. AP, area postrema; BL, basolateral amygdaloid nucleus; BNST, bed nucleus of the stria terminalis; Ce, central amygdaloid nucleus; DMN, dorsomedial hypothalamic nucleus; LH, lateral hypothalamic area; MCPO, magnocellular preoptic nucleus; MD, mediodorsal thalamic nucleus; MPO, medial preoptic nucleus; ox, optic chiasm; PVN, paraventricular hypothalamic nucleus; Pe, periventricular hypothalamic nucleus; PT, paratenial thalamic nucleus; Rt, reticular thalamic nucleus; SM, nucleus of the stria medullaris; SO, supraoptic hypothalamic nucleus; NTS, nucleus of the tractus solitarius; SpVe, spinal vestibular nucleus; VLH, ventrolateral hypothalamic nucleus.

Determining the expression patterns of both receptor and ligand gives key insight into physiological function. *In situ* hybridization histology, RT-PCR, and immunohistochemical studies indicate that PrRP is expressed in neurons of the nucleus tractus solitarius (NTS), the ventrolateral medulla (VLM), and in the caudal portion of the dorsomedial hypothalamic nucleus (DMN) (Figure 1) (Chen et al., 1999; Maruyama et al., 1999; Ibata et al., 2000; Lee et al., 2000). PrRP mRNA has also been found in a number of peripheral tissues, including the adrenal gland, pancreas, placenta, and testis (Fujii et al., 1999; Matsumoto et al., 1999a; Kalliomaki et al., 2004).

The co-localization of PrRP with tyrosine hydroxylase (TH) in the caudal NTS and VLM, suggests that these PrRP cells are a subset of A2 and A1 noradrenergic neurons, respectively (Chen et al., 1999). The highest numbers of PrRP cell bodies are found within the NTS, and interestingly as the hypothalamus shows the highest levels of PrRP fiber immunoreactivity, this suggested the possible projection of PrRP from the brainstem to the hypothalamus (Hinuma et al., 1998; Fujii et al., 1999; Matsumoto et al., 1999a). PrRP-immunoreactive fibers are visible in many areas of the brain, such as the DMN, area postrema (AP), pontine parabrachial area, preoptic areas, bed nucleus of the stria terminalis (BNST), amygdala, mediodorsal nucleus of the thalamus, septal nucleus, and ependymal linings of the ventricles and blood vessels (Lin, 2008). One of the major projection sites is the paraventricular hypothalamus (PVN), where PrRP neurons appear to synapse directly on corticotrophin-releasing hormone (CRH) (Matsumoto et al., 1999a) and oxytocin neurons (Maruyama et al., 1999). Cell-specific connections also have been identified on magnocellular oxytocin/vasopressin neurons of the hypothalamic supraoptic nucleus (Maruyama et al., 1999), somatostatin neurons in the hypothalamic periventricular nucleus (Iijima et al., 2001), and on catecholaminergic cells of the adrenal medulla (Fujiwara et al., 2005).

Distribution of the GPR10 receptor has been investigated using autoradiography, *in situ* hybridization, and RT-PCR (Fujii et al., 1999; Roland et al., 1999; Ibata et al., 2000). The relative level of expression is high in the anterior pituitary, reticular nucleus of the thalamus (Rt), periventricular hypothalamus, DMN, AP, and NTS; with moderate expression in the BNST, PVN, medial preoptic area and nucleus, ventrolateral hypothalamus, stomach, femur, and adrenal gland (Roland et al., 1999).

There is good complementarity in the localization of GPR10 receptor immunoreactive PrRP fiber staining in many brain areas (BNST, supraoptic nucleus, PVN, DMN, and NTS). However, it is interesting to note discrepancies in localization, which might be surprising if GPR10 is the only receptor for PrRP. In fact, many peptide systems have significant mismatches between the distribution of the ligand and their respective cognate receptors. Much of this mismatch might be explained by redundancy in function, that is a receptor will not respond if it is not in contact with the ligand. It may be energetically convenient not to lose the expression of a receptor if there is no evolutionary pressure to do so. Furthermore, peptides often have permissive actions and may not function as classical transmitters at tightly regulated synaptic junctions. For instance, PrRP may be released from neuronal fibers terminating at the ventricular zones, and may enter and diffuse within the cerebral spinal fluid (Iijima et al., 1999); or as seen with substance P, PrRP may diffuse through the neuronal tissue to reach distant receptor sites (Duggan et al., 1990). Although GPR10 is considered to be the cognate receptor for PrRP, others (perhaps currently unknown) may exist. For example, PrRP has significant affinity at neuropeptide FF receptor 2 (NPFF-R2) in *in vitro* studies, and there is potential for overlap between the presence of PrRP and NPFF-R2 particular in the hypothalamus and adrenal gland (Gouarderes et al., 2004). Nevertheless, the diverse distribution profile of receptors and ligand may underlie the diverse physiological roles played by PrRP-GPR10 signaling, and each function needs careful investigation. In the absence of receptor-selective antagonists, this is probably best achieved in receptor knockout mice.

## Role of PrRP in Prolactin Secretion

As high expression of GPR10 is seen in the anterior pituitary, initial studies investigating the physiological action of PrRP focused on hypophysiotropic secretion (Hinuma et al., 1998; Lin et al., 2002b). Preliminary *in vitro* studies, which gave rise to the name of the peptide, described an action of PrRP on prolactin secretion from anterior pituitary tumor cell lines and primary cell cultures (Hinuma et al., 1998). Subsequent studies investigating the relevance of PrRP *in vivo* as a central mediator of prolactin release were controversial, with positive results being reliant on high intravenous PrRP doses administered during specific phases of female rat estrous cycle (Matsumoto et al., 1999b). Other studies demonstrated no prolactin release following central administration of PrRP (Matsumoto et al., 2000; Seal et al., 2002). Moreover, as no PrRP immunoreactivity is found in the median eminence or in hypophysiotropic cells of the hypothalamus (Matsumoto et al., 1999a; Maruyama et al., 2001), classically associated with the secretion of pituitary hormones, the question remains how does PrRP access the pituitary? PrRP may act upon the anterior pituitary as a hormone secreted from peripheral tissues (adrenal, pancreas, testis, placenta), or by an indirect central mechanism possibly via hypophysiotropic neurons (Morales and Sawchenko, 2003). This is a strong possibility, since central administration of PrRP can affect a number of anterior pituitary hormones (Seal et al., 2002). Interestingly, in fish and amphibians, PrRP fibers project to and terminate on prolactin-producing cells of the pituitary and systemic injection of PrRP into rainbow trout causes a release in prolactin and somatolactin (Moriyama et al., 2002; Seale et al., 2002; Sakamoto et al., 2006). PrRP may, therefore, represent an ancient factor for the direct regulation of prolactin secretion that is now evolutionary redundant in this function in higher mammals. Thus, the name, PrRP, may represent a misnomer, as research over the past decade has implicated this signaling pathway in alternative physiological systems.

## Conserved Function of PrRP and GPR10 Signaling in Feeding Behavior

Prolactin-releasing peptide belongs to the RFamide neuropeptide family (Osugi et al., 2006). Although this family impacts on a diverse range of physiological functions, almost all have been shown to modulate food intake (Bechtold and Luckman, 2007). This involvement of the RFamides in feeding behavior has been demonstrated across most animal taxa, including coelenterates, mollusks, amphibians, birds, and mammals, suggesting an evolutionary conserved role in energy homeostasis (Dockray, 2004).

Numerous studies suggest a pivotal role of PrRP in the homeostatic regulation of feeding and energy balance. Evidence from our group has shown that central administration of PrRP decreases feeding and body weight gain in rats and mice without causing adverse effects (Lawrence et al., 2000, 2002; Bechtold and Luckman, 2006), and that PrRP mRNA in the DMN, NTS, and VLM is downregulated in states of negative energy balance (Lawrence et al., 2000). Importantly, these central anorexic actions of PrRP are not present in mice (Bechtold and Luckman, 2006) or rats (Watanabe et al., 2005) that lack functional expression of GPR10, highlighting the significance of endogenous PrRP-GPR10 signaling in food intake. The significance of this system to energy homeostasis generally is validated further by the obese and hyperphagic phenotypes of both PrRP^−/−^ and GPR10^−/−^null mice (Gu et al., 2004; Takayanagi et al., 2008).

As PrRP induces hypophagia without evoking a conditioned taste aversion or disrupting the normal behavioral satiety sequence (Lawrence et al., 2002), it seems likely that PrRP-GPR10 signaling plays an integral part of the brain’s endogenous appetitive neurochemistry. In fact, PrRP induces a significant temporal advancement in the behavioral satiety sequence, an affect associated with natural satiety factors like cholecystokinin-8 (CCK) (Lawrence et al., 2002). Furthermore, experiments with PrRP^−/−^mice or PrRP-neutralizing antibodies, in the laboratory of Tatsushi Onaka, show that PrRP regulates meal size rather than meal frequency, indicating that PrRP may mediate appetite by direct actions on satiation (Takayanagi et al., 2008). As the brainstem medulla oblongata and, in particular, the NTS receives extensive gastrointestinal vagal inputs, these PrRP neurons are an obvious candidate for a role in gut-brain signaling. CCK is released from enteroendocrine cells in response to a meal, and acts via the CCK_1_ receptor on vagal afferent neurons which terminate in the NTS (Saper, 2004). PrRP neurons localized in both the NTS and the VLM show strong functional activation in response to anorexic doses of CCK (Lawrence et al., 2002). Central administration of PrRP elicits a similar pattern of neuronal c-Fos protein expression as that observed following intraperitoneal administration of CCK (Luckman, 1992; Lawrence et al., 2002; Bechtold and Luckman, 2006), and the anorexic effects of CCK are impaired in both PrRP ^−/−^ and GPR10^−/−^null mice (Bechtold and Luckman, 2006; Takayanagi et al., 2008).

The downstream actions of PrRP neurons within the brainstem remain to be clarified. However, PrRP receptor is present in the dorsal vagal complex (Roland et al., 1999; Ibata et al., 2000) and PrRP can act pre-synaptically to affect the firing of preganglionic vagal efferents involved in regulating gut function (Morales and Sawchenko, 2003). Thus, although not proven, it is likely that PrRP-GPR10 signaling within the dorsal vagal complex may mediate the effects of CCK on the parasympathetic regulation of gut motility and secretion. The sensation of satiety, and integration with descending motor pathways to regulate feeding, requires integration with higher brain centers. PrRP-immunoreactive fibers and GPR10 mRNA expression have been demonstrated in a number of hypothalamic nuclei (Fujii et al., 1999; Maruyama et al., 1999; Roland et al., 1999; Ibata et al., 2000; Lee et al., 2000). In particular, PrRP-containing neurons in the NTS project directly to the PVN (Onaka, 2004), where neurons containing CRH or oxytocin possess PrRP receptor (Lin et al., 2002a; Takayanagi and Onaka, 2010). Though anorexic doses of PrRP activate neurons expressing CRH or oxytocin in the PVN (Bechtold and Luckman, 2006; Mera et al., 2006), it is difficult to relate this specifically to satiety signaling, as these neurons may equally be involved in responses to stress (see below). However, PrRP-induced anorexia is attenuated by CRH receptor antagonists (Bechtold and Luckman, 2006), while oxytocin receptor antagonists attenuate the anorexic actions of both PrRP and CCK (Olson et al., 1991; Blevins et al., 2003). Further work will be required to dissect the relative importance of ascending PrRP pathways on satiety and stress-related stimuli. Additional consideration for the role of DMN PrRP neurons in the regulation of feeding behavior is needed also. However, our working model is that PrRP-GPR10 signaling mediates the CCK-vagal regulation of gut function following a meal at the level of the dorsal vagal complex in the brainstem. Further integration with higher brain centers is achieved through the projection of PrRP neurons to the hypothalamus. This may include CRH and oxytocin neurons of the PVN, the latter, at least, having an accepted role in the descending fine regulation of the dorsal vagal complex and feeding control (Samson et al., 2000; Yamada et al., 2009; Onaka et al., 2010).

## Energy Homeostasis

Though the evidence for PrRP-GPR10 functioning in satiation is strong, this does not infer a role in overall energy balance, and an interaction with other metabolic regulators might be expected. We have shown that the expression of PrRP is down regulated in situation where the animal is in real (e.g., fasting or lactation) or in perceived (e.g., Zucker rat) negative energy balance (Ellacott et al., 2002). That is, situations which correlate with reduced leptin signaling. Leptin is an adipose-derived hormone, that signals levels of peripheral fat storage to the brain to regulate long-term metabolism (Denver et al., 2011). Immunohistochemical studies have suggested that PrRP neurons (and TH-positive cells) in the brainstem and hypothalamus of the rat express leptin receptors and, thus, that there is a direct cellular effect of the hormone (Hay-Schmidt et al., 2001; Ellacott et al., 2002). However, a more recent paper failed to co-localize leptin receptor in brainstem PrRP neurons of the mouse (Garfield et al., 2012). Leptin induces the expression of phosphorylated signal transducer and activator of transcription protein 3 (pSTAT3) in PrRP neurons, especially those in the DMH (Takayanagi et al., 2008). Central co-administration of PrRP and leptin results in augmented hypophagia and body weight loss (Ellacott et al., 2002), and the hypophagic effects of leptin are impaired in PrRP^−/−^ (Takayanagi et al., 2008) and GPR10^−/−^ null mice (our unpublished results). PrRP-GPR10 clearly has a role in the response to leptin, but whether this is due to a direct or indirect effect of leptin on PrRP neurons remains to be determined.

The maintenance of energy homeostasis involves the balance of both energy intake and energy expenditure. Interestingly pair-feeding studies indicate that the reduced weight gain measured in rats treated with PrRP is not accounted for solely by a reduction in food intake, suggesting that PrRP also affects energy expenditure (Lawrence et al., 2000, 2004). PrRP administration acutely increases body temperature, O_2_ consumption, and UCP-1 expression of brown adipose tissue in rats (long before any effect on body weight), suggesting direct modulation by PrRP of energy expenditure (Lawrence et al., 2004). Furthermore, GPR10^−/−^ knockout mice exhibit a much lower basal metabolic rate, when compared with wild-type mice (our unpublished data), which likely contributes to the obese phenotype of these animals (Gu et al., 2004). Thus, PrRP-GPR10 signaling can induce energy expenditure and thermogenesis, which is interesting considering the known role of the DMN in thermoregulation (Willette et al., 1984; Aicher et al., 1995; Horiuchi et al., 2002). In addition, PrRP may play a role in mediating energy consumption under stressful conditions, as the increase oxygen consumption seen in response to stressful stimuli is attenuated in PrRP^−/−^ mice (Onaka et al., 2010).

## Roles of PrRP and GPR10 Signaling in the Control of Stress Responses

Brain nuclei expressing PrRP and GPR10, such as in the medulla oblongata and the hypothalamus, have been implicated in mediating stress responses (Onaka, 2004). PrRP neurons within these regions respond to a variety of stressful stimuli including body restraint, fear conditioning (Zhu and Onaka, 2003), footstock, hemorrhage (Uchida et al., 2010), and inflammatory stress (Mera et al., 2006). PrRP neurons may, therefore, play an important role in the neuroendocrine response to stress.

Retro-grade tracing of the PrRP neurons innervating the PVN indicates that the fibers originate within the VLM and NTS, where they co-localizes with noradrenaline in the A1 and A2 neuronal populations, respectively (Chen et al., 1999; Minami et al., 1999; Roland et al., 1999; Morales et al., 2000; Maruyama et al., 2001). These noradrenergic neurons are well known mediators of stress in the central nervous system. Models of emotional stress, including conditioned fear stimulation and water immersion/restraint activate medullary PrRP neurons and increases PrRP mRNA expression (Maruyama et al., 2001; Morales and Sawchenko, 2003; Zhu and Onaka, 2003). Interestingly, PrRP and noradrenaline, which co-localize in A1/A2 cells, act synergistically to induce systemic ACTH release (Maruyama et al., 2001).

One way in which PrRP may influence stress response is by the dense network of PrRP clustered on CRH and oxytocin neurons in the PVN and BNST (Iijima et al., 1999; Maruyama et al., 1999; Ibata et al., 2000). Central administration of PrRP dramatically increases c-Fos expression in CRH neurons in the PVN, an effect that results in the concomitant release of ACTH, oxytocin, and corticosterone into the systemic circulation (Matsumoto et al., 2000; Seal et al., 2002). Importantly, blockade of endogenous PrRP signaling by administration of PrRP neutralizing antibodies attenuates stress induced activation of PVN neurons and reduces systemic oxytocin release (Zhu and Onaka, 2003; Mera et al., 2006).

Although contacts are seen between PrRP fibers and CRH neurons in the PVN (Matsumoto et al., 2000), their relative paucity suggests that these synapses are unlikely to be responsible for the entire modulation of CRH neurons in the PVN. Double *in situ* hybridization shows that the majority of cells expressing GPR10 in the PVN are in fact CRH-negative, whereas GPR10 is co-expressed extensively with CRH in the BNST (Lin et al., 2002b). The BNST not only receives extensive PrRP nerve fibers (Maruyama et al., 1999), it is involved with stress responses via a direct modulation of the PVN (Palkovits et al., 1980; Lin et al., 2002b). It, therefore, seems possible that PrRP may also regulate CRH neurons in the PVN indirectly via the BNST. These results suggest that whether directly or indirectly, PrRP is a potent stimulator of CRH neurons in the PVN, inferring access to the hypothalamic–pituitary–adrenal axial control of stress.

Stressful stimuli affect food intake and energy expenditure, while food intake and energy expenditure affect stress responses (Kawakami et al., 2008). For instance PrRP^−/−^ mice show a reduced increase in oxygen consumption following stressful stimuli (Onaka et al., 2010). It seems tempting to suggest that PrRP may modulate food intake in times of stress. Although only speculative further examination of this hypothesis using conditional transgenic mice for PrRP and GPR10 could help shed light on this theory.

## Effects of PrRP and GPR10 on Blood Pressure

Central injection of PrRP results in a significant increase in blood pressure and cardiovascular output in conscious, unrestrained rats (Samson et al., 2000). Numerous studies describe integral roles played by the NTS, AP, and VLM in mediating cardiovascular function (Yamada et al., 2009). It seems likely that PrRP neurons in these regions may be involved. The NTS and AP receive visceral and hormonal information from peripheral cardiovascular sites (Aicher et al., 1995), so PrRP and GPR10 in these sites may be in a position to modify the ascending and descending efferent connections mediating blood pressure homeostasis (Willette et al., 1984; Aicher et al., 1995). Site specific administration of PrRP directly into the caudal VLM (where a population of PrRP neurons are localized) results in a dose-dependent increase in mean arterial blood pressure, heart rate, and renal sympathetic activity (Horiuchi et al., 2002). Interestingly however, PrRP has no effect when injected directly into the rostral VLM, AP, or the NTS. How PrRP modulates blood pressure homeostasis in an area of minimal GPR10 receptor expression such as the VLM and not in regions of high receptor expression such as the AP and NTS remains enigmatic (Chen et al., 1999; Roland et al., 1999).

Although the mechanisms underlying the pressor effects of PrRP are undefined, a recent study by Yamada et al. (2009) suggests the involvement of CRH neurons in the PVN. PrRP neurons from the VLM project to the PVN where they synapse on CRH positive cells. Central CRH has a known effect of elevating blood pressure in response to stressors (i.e., CRH stimulates sympathetic nerves via the CRH_1_ receptor) (Vale et al., 1983; Spina et al., 2000). Yamada et al. (2009) show that pressor- and tachycardia-inducing doses of PrRP activate oxytocin-, vasopressin-, and CRH-producing neurons in the PVN. Furthermore, the elevation of blood pressure and heart rate elicited by PrRP administration are completely suppressed by treatment with a CRH antagonist. PrRP neurons in the VLM may, therefore, mediate CRH release to regulate the cardiovascular system via the sympathetic nervous system.

Finally, the receptor mediating PrRP pressor and tachycardia effects remains unclear. Epidemiological human studies show an association of polymorphisms in the GPR10 receptor with blood pressure, thus implying a potential role of the GPR10 receptor in blood pressure regulation (Bhattacharyya et al., 2003). Contrastingly, PrRP can still elicit effects on mean arterial blood pressure and heart rate in Otsuka Long-Evans Tokushima Fatty (OLETF) rat strain, in which the GRP10 receptor gene is naturally mutated (Ma et al., 2009). Instead, PrRP effects were blocked by administration of the NPFF-2R antagonist, RF9, suggesting that PrRP may modulate blood pressure homeostasis via the NPFF-2R (Ma et al., 2009). It will be useful to follow up these studies using other models since neither the OLETF rat (which has at least one other natural mutation, in the CCK_1_ receptor gene), nor the R9 antagonist, are the best tools available. Certainly, if unwanted cardiovascular effects of PrRP are mediated solely by the NPFF-R2, there could be therapeutic potential for selective GPR10 agonists as drug targets other metabolic diseases.

## Effects of PrRP and GPR10 Circadian Rhythmicity and Sleep Regulation

The expression of GPR10 in particular brain regions, including the preoptic area, the histaminergic ventral tuberomammillary nucleus, the noradrenergic locus ceruleus, serotonergic dorsal raphe, and suprachiasmatic nucleus suggested that PrRP-GPR10 signaling may play a part in circadian rhythmicity and/or sleep regulation (Chen et al., 1999; Roland et al., 1999). The relative importance of PrRP-GPR10 signaling in each of these specific nuclei is yet to be investigated, however, a wealth of literature exists implicating an integral role in sleep and arousal (for reviews, see Suntsova et al., 2009; Szymusiak, 2010; Brown et al., 2012; Murillo-Rodriguez et al., 2012). One region of particular interest, which has a high GPR10 receptor expression, is the Rt (Roland et al., 1999). The Rt is predominantly GABAergic and acts as a gateway for ascending inputs into the cortex that regulate the transition into sleep (Steriade, 2005; Timofeev and Chauvette, 2011). Central administration of PrRP is known to modulate sleep oscillation, and promote rapid and prolonged arousal (Zhang et al., 2000; Lin et al., 2002a). Furthermore, electrophysiological experiments on brain slices show that administration of PrRP attenuates oscillatory activity generated in the Rt, a phenomena that could underlie PrRP’s modulation of circadian and sleep regulation (Lin et al., 2002a).

## Future Perspectives

Since the de-orphanization of GPR10, research into the physiological roles of PrRP neurotransmission has been varied and exciting. The PrRP peptide is conserved among species (fish, amphibians, birds, and mammals), pointing toward it seems both primitive and important function (Dockray, 2004; Bechtold and Luckman, 2007). Research into the physiological roles of PrRP has evolved from the initial observations as a “PrRP” (perhaps resulting in a misnomer) to a multifunctional protein integral to a number of functions. Given the current understanding of the PrRP-GPR10, it seems likely that this ancient signaling system may act in times of stress to regulate feeding behavior, induce energy expenditure and increased cardiac output, heighten arousal, and allow the systemic release of endocrine factors. It is possible that, in mammalian species, some of these functions have been modified more specifically, for example into a role in satiation and energy regulation.

To further examine the importance of PrRP-GPR10 signaling a number of outstanding questions need to be addressed.

### What are the relative importance of the different populations of PrRP-producing cells in the brainstem, hypothalamus, and in peripheral tissues?

This review has highlighted potential differential roles for the PrRP-expressing neuronal populations. Although currently only speculative, it seems that the VLM may play a specific role in the pressor effects of PrRP (Horiuchi et al., 2002); whereas, the NTS appears important in mediating CCK’s effect on satiation (Bechtold and Luckman, 2006). Interestingly, a recent study has shown that systemic CCK acts to attenuate liver gluconeogenesis independently of insulin production (Cheung et al., 2009). Importantly, the effect requires the integration of a gut–brain–liver axis; effects that could be centrally mediated by CCK responsive PrRP neurons in the NTS.

Further speculation arises over the function of the DMN PrRP population. Takayanagi and Onaka (2010) show significant pSTAT3 co-expression following leptin administration in DMN, suggesting that DMN PrRP neurons are responsive to leptin. Although there is little doubt of the importance of leptin receptor in energy homeostasis, recent research has specifically identified leptin responsive neurons in the DMN as mediators of adaptive thermogenesis (Enriori et al., 2011; Bechtold et al., 2012). As adaptive thermogenesis and the neuronal circuitry innervating brown adipose tissue is currently topical in the domain of anti-obesity therapeutics, it is important to investigate whether the PrRP neurons play a part. Also, both PrRP and GPR10 are expressed in peripheral tissues (Roland et al., 1999). Nothing is known about the importance of peripheral PrRP-GPR10 signaling, and the exploration of these interactions could be vital to the development of viable GPR10 therapeutics.

The recent advancements in the generation of conditional transgenic mice, makes finding the answers to these questions possible. For instance the generation of mice conditionally expressing PrRP under the control of different promoters will allow the genetic dissection of specific populations.

### What are the downstream and upstream targets of PrRP – GPR10 signaling?

Understanding the neurochemical make up of PrRP target neurons will help to further define and dissect out the relative importance of each neuronal population. Recent advancements in antero- and retro-grade labeling using Cre recombinase specific adenoviral vectors could advance our understanding of how specific PrRP neuronal population integrate into both the local and global neuronal circuits (Gautron et al., 2010).

There is as a potential caveat with much of the work already achieved in understanding PrRP-GPR10 signaling. As mentioned above, there is some divergence, at least in the mammalian system, between PrRP-expressing nerve fibers and the location of GPR10 receptors. Thus, an element of deliberation must be taken to understanding studies whereby PrRP is administered globally into the brain and at non-physiological doses, as this could be activating redundant receptors. Although this problem may be minimal, the use of conditional transgenic animals, conditional viral vectors, and the development of selective GPR10 agonists/antagonists will greatly enhance our understanding of this important neurotransmitter system. Although much of the ground work has been established, there is still much to learn about PrRP-GPR10 signaling and its definitive roles in the nervous system.

## Conflict of Interest Statement

The authors declare that the research was conducted in the absence of any commercial or financial relationships that could be construed as a potential conflict of interest.

## Abbreviations

7TMR, seven-transmembrane-domain receptors; ACTH, adrenocorticotropic hormone; AP, area postrema; BL, basolateral amygdaloid nucleus; BNST, bed nucleus of the stria terminalis; CCK, cholecystokinin-8; Ce, central amygdaloid nucleus; DMN, dorsomedial hypothalamic nucleus; GABA, γ-aminobutyric acid; GPCR, G-protein-coupled receptors; GPR10, G-protein-coupled receptor 10; LH, lateral hypothalamic area; MCPO, magnocellular preoptic nucleus; MD, mediodorsal thalamic nucleus; MPO, medial preoptic nucleus; ox, optic chiasm; NPFF-R2, neuropeptide FF receptor 2; NPY, neuropeptide Y; NTS, nucleus of the tractus solitarius; OLETF, Otsuka Long-Evans Tokushima Fatty; Pe, periventricular hypothalamic nucleus; PrRP, prolactin-releasing peptide; PT, paratenial thalamic nucleus; PVN, paraventricular hypothalamic nucleus; Rt, reticular nucleus of the thalamus; SM, nucleus of the stria medullaris; SO, supraoptic hypothalamic nucleus; SpVe, spinal vestibular nucleus; TH, tyrosine hydroxylase; VLH, ventrolateral hypothalamic nucleus; VLM, ventrolateral medulla.
